# Plasma Rivaroxaban Level to Identify Patients at Risk of Drug Overexposure: Is a Single Measurement of Drug Level Reliable?

**DOI:** 10.1055/s-0040-1721734

**Published:** 2021-02-25

**Authors:** Krishnan Shyamkumar, Jack Hirsh, Vinai C. Bhagirath, Jeffrey S. Ginsberg, John W. Eikelboom, Noel C. Chan

**Affiliations:** 1 Population Health Research Institute, Hamilton, Ontario, Canada; 2 Division of Hematology and Thromboembolism, Department of Medicine, McMaster University, Hamilton, Ontario, Canada; 3 Thrombosis and Atherosclerosis Research Institute, Hamilton, Ontario, Canada

**Keywords:** rivaroxaban, drug levels, NOACs, atrial fibrillation, venous thromboembolism

## Abstract

**Introduction** Dose adjustment based on laboratory monitoring is not routinely recommended for patients treated with rivaroxaban but because an association has been reported between high drug level and bleeding, it would be of interest to know if measuring drug level once could identify patients at risk of bleeding who might benefit from a dose reduction.

**Objective** This study was aimed to investigate the reliability of a single measurement of rivaroxaban level to identify clinic patients with persistently high levels, defined as levels that remained in the upper quintile of drug-level distribution.

**Methods** In this prospective cohort study of 100 patients with atrial fibrillation or venous thromboembolism, peak and trough rivaroxaban levels were measured using the STA-Liquid Anti-Xa assay at baseline and after 2 months. Values of 395.8 and 60.2 ng/mL corresponded to the 80th percentile for peak and trough levels, respectively, and levels above these cut-offs were categorized as high for our analyses.

**Results** Among patients with a peak or trough level in the upper quintile at baseline, only 26.7% (95% confidence interval [CI]: 10.9–52.0%), and 13.3% (95% CI: 2.4–37.9%), respectively, remained above these thresholds.

**Conclusion** Our findings do not support the use of a single rivaroxaban level measurement to identify patients who would benefit from a dose reduction because such an approach is unable to reliably identify patients with high levels.

## Introduction

Based on their convenience and favorable results in clinical trials, the direct oral anticoagulants (DOACs) are replacing vitamin-K antagonists for various clinical indications. 1 2 Rivaroxaban was the first oral factor Xa inhibitor licensed for stroke prevention in atrial fibrillation (AF) and for venous thromboembolism (VTE) prevention and treatment. 3 Like the other DOACs, drug levels of rivaroxaban vary among patients and there is indirect evidence from a subanalysis of the ROCKET-AF trial that the risk of bleeding correlates with rivaroxaban levels as measured by the prothrombin time. 4 Based partly on these data, it has been suggested that measuring drug levels would be useful for identifying patients who might benefit from a dose reduction. 5 6 7 However, dose adjustment based on drug level measurement would only be practical if the dose response to rivaroxaban did not vary over time. In this report, we examine the possibility that an approach based on a single random measurement of peak or trough rivaroxaban level can be used to identify patients with persistently high levels who might benefit from a dose reduction.

## Methods

### Study Design

Present study is a prospective observational study of 100 patients. The study protocol was reviewed and approved by the Hamilton Integrated Research Ethics Board.

### Patients

Consecutive adult patients with AF (permanent, paroxysmal, or persistent) or VTE (idiopathic or provoked) receiving long-term rivaroxaban therapy were enrolled from outpatient clinics at the Hamilton General Hospital. Eligible patients who were either geographically inaccessible for follow-up, or unwilling or unable to provide written informed consent, were excluded.

### Follow-up

Patients were seen at baseline and 2 months after enrolment with trough and peak rivaroxaban levels measured at each visit. As this was a noninterventional study to evaluate inter- and intraindividual variability of rivaroxaban levels with the current dosing scheme, clinicians were blinded to the results of these measurements.

### Collection and Processing of Blood Samples

Attempts were made to measure rivaroxaban level at steady state. This meant that patients had to be receiving once daily rivaroxaban for at least 1 week before blood sampling, and for patients with VTE, we included only levels measured when treated with rivaroxaban 20-mg daily. Blood samples for obtaining trough drug levels were collected 24 hours after the last dose of rivaroxaban. Patients were then directed to take their next daily dose, and blood samples for obtaining peak drug levels were collected 2.5 hours later. At each time point, 10 mL of blood was collected into Becton Dickinson Vacutainer tubes (Becton Dickinson, Mississauga, Ontario, Canada), containing 3.2% buffered trisodium citrate (9:1, vol/vol) by a trained research assistant. Immediately after collection, the tube was inverted three to five times, cellular elements were sedimented by twice subjecting the sample to centrifugation at 1,700× *g* for 15 minutes at 23°C, and the resultant platelet-poor plasma was then harvested and stored in 1-mL aliquots at −80°C.

### Antifactor Xa Activity Assay

Plasma anti-Xa activity was measured using the STA-Liquid Anti-Xa assay as per the manufacturer's instructions, on the STA-R platform (Diagnostica Stago, Ansières-sur-Seine, France). Rivaroxaban concentration was estimated by referring to a calibration curve constructed with rivaroxaban calibrators (Diagnostica Stago, Ansières-sur-Seine, France). The limit of detection was 30 ng/mL, and this value was imputed for the purpose of statistical analysis, whenever patients had levels below the limit. The inter- and intra-assay coefficients of variation (CVs) are reported to be 1.0 to 4.3 and 0.8 to 3.3%, respectively, lower than the expected intra- and interpatient CVs of rivaroxaban level. 8

### Statistical Analyses

Descriptive statistics were used to summarize the data. Normally distributed data were described using means and standard deviation if normally distributed and median and interquartile range if not. Categorical data were reported as proportions with 95% confidence intervals (CIs), calculated using the Wald method. To assess the reliability of a single measurement to identify patients with a high rivaroxaban level, we use the 80th percentile of baseline peak and trough drug level distribution as cut-offs and calculate the proportions (and 95% CI) of patients whose levels remained high at the subsequent visit. Values of 395.8 and 60.2 ng/mL corresponded to the 80th percentile for peak and trough levels, respectively, and levels above these cut-offs were categorized as high for our analyses. Intrapatient variability of rivaroxaban in patients with an initial high drug levels was further explored by examining the proportions (and 95% CIs) of drug levels that fell below the median level on subsequent measurements. These analyses were performed independently for peak and trough levels.

## Results

**Fig. 1:**
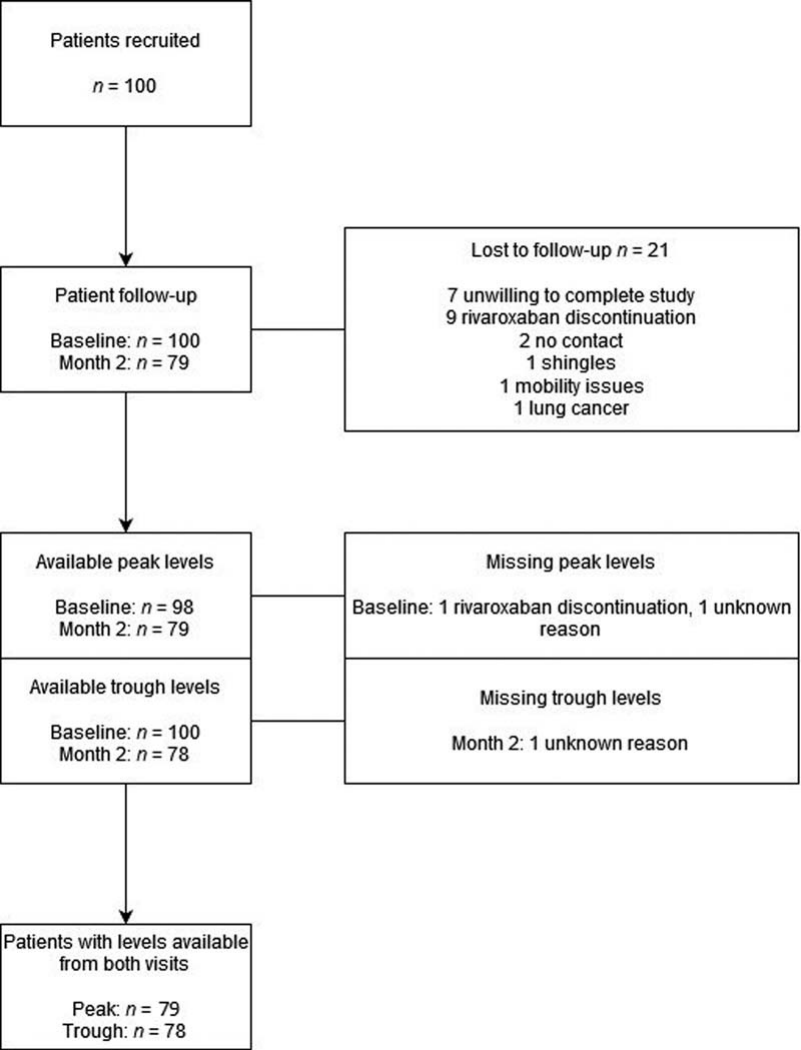
Patient recruitment, follow-up and availability of test results.

Fig. 1 summarizes the follow-up of recruited patients and the number of plasma rivaroxaban levels available for analysis at each visit. Of the 100 patients enrolled, 10 patients were unwilling or unable to attend the second visit, 9 had rivaroxaban discontinued, and 2 could not be contacted for the follow-up visit. A total of 79 patients with levels measured at both visits were included in the intrapatient variability analysis. Results for peak plasma levels were available at baseline and month 2 for 98 and 79 patients, respectively, and those for trough levels were available for 100 and 78 patients, respectively. Blood for trough levels was collected at a median (interquartile range [IQR]) of 24.8 (23.8–25.8) hours while blood for peak levels was collected at 2.5 (2.4–2.5) hours after rivaroxaban ingestion.

### Baseline Characteristics

The baseline characteristics of the entire cohort and the analysis population (i.e., the subgroup of patients for whom drug levels were measured at both baseline and month 2) are presented in Table 1 . The mean age and weight of the entire cohort were 64.7 years and 90.1 kg, respectively, and the median creatinine clearance was 97.9 mL/min. About 64% of these patients were male, and the proportion of patients taking rivaroxaban for AF was 51%. The baseline characteristics of the analysis population were similar to those of the entire cohort.

**Table 1:** Baseline characteristics of the entire study population and the analysis population

	All patients ( *n* = 100)	Analysis population ( *n* = 79) a
Age (y) Mean ± SD	64.7 ± 13.0	65.4 ± 12.3
Male sex *n* (%)	64 (64.0)	50 (63.3)
Weight (kg) Mean ± SD	90.1 ± 20.5	90.2 ± 20.9
Weight 50–100 kg *n* (%)	75 (75.0)	59 (74.7)
Weight > 100 kg *n* (%)	25 (25.0)	20 (25.3)
CrCl (mL/min) Median (range)	97.9 (32.0–283.5)	94.5 (32.0–206.4)
Indications *n* (%)		
Atrial fibrillation	51 (51.0)	40 (51.3)
Venous thromboembolism	49 (49.0)	39 (48.7)
Dosing *n* (%)		
20 mg once daily	91 (91.0)	75 (94.9)
15 mg once daily	9 (9.0)	4 (5.1)

Abbreviations: CrCl: creatinine clearance estimated using the Cockcroft–Gault formula; SD, standard deviation.

a Analysis population: patients with measurements of drug levels at both visits.

### Ranges for Peak and Trough Levels

For the entire population, median peak levels (p10 and p90) at baseline and month 2 were 280.5 (159.6–419.56) and 252 (127–458) ng/mL, respectively, whereas median trough levels (p10 and p90) at baseline and month 2 were 43.2 (28.1–90) and 35.5 (25–62.1) ng/mL, respectively. Among patients with rivaroxaban measurements performed at both visits, the median peak levels (p10 and p90) at baseline and month 2 were 278 (159.6–419.3) and 251 (126.8–460) ng/mL, respectively, and the corresponding trough levels (p10 and p90) were 43 (29.5–91.3) and 35.5 (25–62.1) ng/mL, respectively.

### Intrapatient Variability of Rivaroxaban in Patients with High Drug Levels

#### Peak levels

**Fig. 2:**
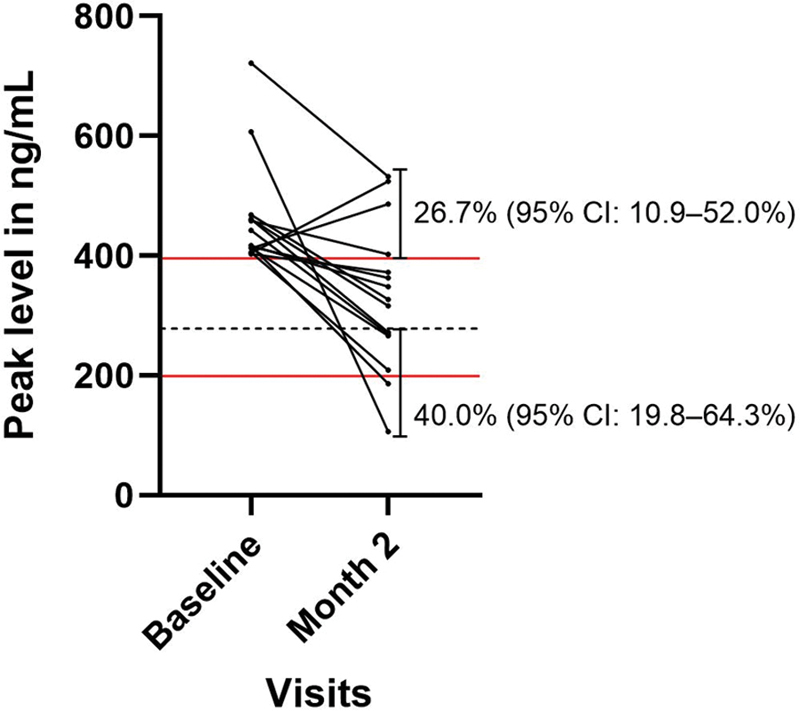
Change in peak rivaroxaban levels across visits in patients with levels falling in the upper 20th percentiles at baseline (≥395.8 ng/mL). Solid, red horizontal lines represent the 20th and 80th percentiles of peak levels observed at baseline. Dashed, black horizontal lines represent the median peak level at baseline (278.0 ng/mL). Proportions of patients with levels remaining above or below the extreme 20th percentiles at month 2 are shown by the respective vertical capped lines.

Fig. 2 shows the change in peak level over time in patients whose initial levels fell in the upper quintile of the drug level distribution (≥395.8 ng/mL). Of these patients, only 26.7% (95% CI: 10.9–52.0%) had levels that remained above this threshold, and approximately 40.0% (95% CI: 19.8–64.3%) had levels that fell below the median peak level at month 2 ( Table 2 ).

**Table 2:** Intraindividual variability of rivaroxaban levels in patients with an initial high level

	Peak levels ^a^	Trough levels ^b^
No of patients (%) with persistently high levels on repeat measurement	4/15 26.7% (95% CI: 10.9–52.0%)	2/15 13.3% (95% CI: 2.4–37.9%)
No of patients (%) with levels below the median c on repeat measurement	6/15 40.0% (95% CI: 19.8–64.3%)	5/15 33.3% (95% CI: 15.2–58.3%)

Abbreviation: CI, confidence interval.

Values of ^a^ 395.8 and ^b^ 60.2 ng/mL corresponded to the 80th percentile for peak and trough levels, respectively, and levels above these cut-offs were categorized as high for our analyses.

c Median peak and trough levels were 278.0 and 43.0 ng/mL, respectively.

#### Trough Levels

**Fig. 3:**
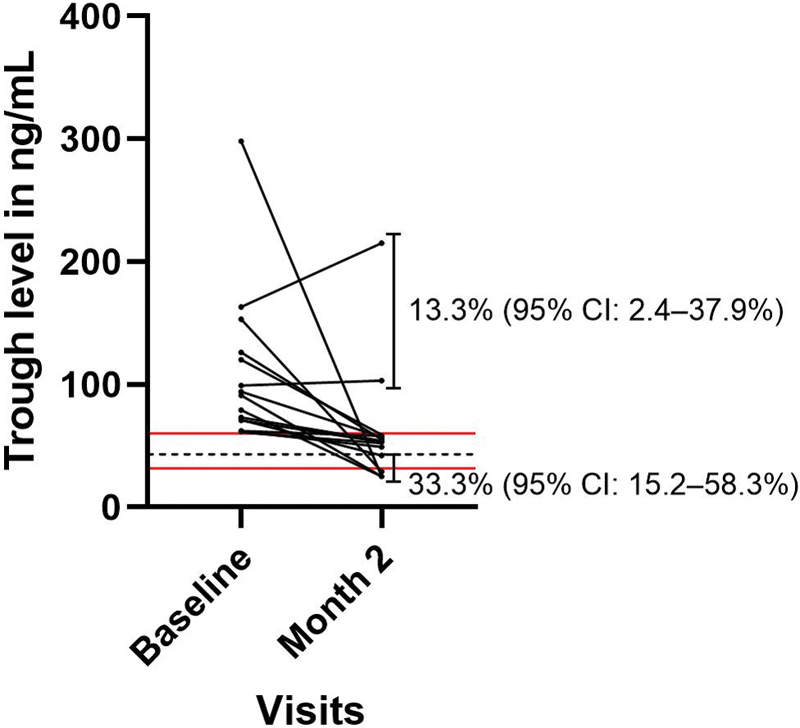
Change in trough rivaroxaban levels across visits in patients with levels above the upper 20th centiles at baseline (≥60.2 ng/mL). Solid, red horizontal lines represent the 20th and 80th percentiles of peak levels observed at baseline. Dashed, black horizontal lines represent the median trough level at baseline (43.0 ng/mL). Proportions of patients with levels remaining above or below the extreme 20th percentiles at month 2 are shown by the respective vertical capped lines. CI, confidence interval.

Fig. 3 shows the change in trough level over time in patients whose initial levels fell in the upper quintile of the distribution (≥60.2 ng/mL). Of these patients, 13.3% (95% CI: 2.4–37.9%) had levels that remained above this threshold, while 33.3% (95% CI: 15.2–58.3%) had levels that fell below the median baseline level at month 2 ( Table 2 ).

## Discussion

We examined the possibility that an approach that measures rivaroxaban level once can be used to identify patients with persistently high rivaroxaban levels. We showed that at least two-thirds of patients with an initial peak or trough levels that fell above the respective 80th percentiles had subsequent levels that no longer fell in these extremes. The findings suggest that the measurement of a single peak or trough drug level cannot be used to reliably identify patients with persistently high rivaroxaban levels who might benefit from dose reduction.

Other studies have reported moderate intrapatient variability in rivaroxaban level based on an estimation of the CV of the study population but such measure is difficult to interpret clinically and does not directly apply to patients with extreme levels. 8 9 10 11 12 Our findings are, however, consistent with those of Gulpen and colleagues who explored the intrapatient variability in postdose rivaroxaban level over 1 year using a similar approach to ours. 13 Like us, they showed that a high proportion (∼60%) of patients with an initial level that fell in the upper quintile subsequently had levels that were no longer high, but there were also some who had persistently high levels. Gulpen and colleagues concluded that their finding of a significant proportion of patients with persistently high or low drug levels offers the opportunity to adjust doses or switch to a different DOAC to enhance safety and efficacy of DOAC treatment. 13 However, identification of patients potentially suitable for dose adjustment would require serial measurement of blood levels and before being accepted the efficacy and safety of such an approach would have to be demonstrated in a randomized trial.

Our findings suggest that a strategy based on single peak or trough rivaroxaban level measurement is not reliable to identify patients who should undergo a dose reduction. Furthermore, they raise the possibility for harm if the dose is reduced on the basis of one measurement because, on repeat testing, up to one-third of our patients had subsequent levels that were lower that the median drug level.

## Strengths and Limitations

Our study has strengths and limitations. We carefully timed specimen collection to minimize preanalytical variability and examined variability in both trough and peak levels in patients with high drug levels. Potential limitations include the use of only one assay type to measure rivaroxaban levels, and the attrition of patients over time (∼20%), which may have reduced the precision of our estimates, although these findings highlight the practical challenges associated with patient adherence to a routine monitoring strategy. In addition, our study did not examine the determinants of variability in rivaroxaban level. Finally, our findings cannot exclude the possibility that multiple drug level measurements might identify patients who would benefit from a dose reduction. However, the net clinical benefit of such an approach requires further evaluation in randomized trials.

## Conclusion

Our findings do not support the use of a single rivaroxaban level measurement to identify patients who would benefit from a dose reduction because such an approach is unable to reliably identify patients with high levels.
